# Rational Engineering of the Substrate Specificity of a Thermostable D-Hydantoinase (Dihydropyrimidinase)

**DOI:** 10.3390/ht9010005

**Published:** 2020-02-12

**Authors:** Hovsep Aganyants, Pierre Weigel, Yeranuhi Hovhannisyan, Michèle Lecocq, Haykanush Koloyan, Artur Hambardzumyan, Anichka Hovsepyan, Jean-Noël Hallet, Vehary Sakanyan

**Affiliations:** 1 SPC Armbiotechnology, National Academy of Sciences of Republic of Armenia, Yerevan 0056, Armenia; haganyants@gmail.com (H.A.); eranuhi.hov@gmail.com (Y.H.); ankoloyan@gmail.com (H.K.); arthambardzumyan@gmail.com (A.H.); anichka_h@yahoo.com (A.H.); 2 UFIP CNRS 6286, Faculté de Biologie, Université de Nantes, 44322 Nantes, France; pierre.weigel@univ-nantes.fr (P.W.); jean-npel.hallet@univ-nantes.fr (J.-N.H.); 3 IICiMed, Faculté de Pharmacie, Université de Nantes, 44035 Nantes, France; michelle.lecocq@univ-nantes.fr; 4 IICiMed, Faculté de Biologie, Université de Nantes, 44332 Nantes, France; 5 ProtNeteomix, 44332 Nantes, France

**Keywords:** D-hydantoinase, dihydropyrimidinase, substrate specificity, manganese-dependence, site-directed mutagenesis, thermophilic bacteria, long inverse PCR, biocatalyst

## Abstract

D-hydantoinases catalyze an enantioselective opening of 5- and 6-membered cyclic structures and therefore can be used for the production of optically pure precursors for biomedical applications. The thermostable D-hydantoinase from *Geobacillus stearothermophilus* ATCC 31783 is a manganese-dependent enzyme and exhibits low activity towards bulky hydantoin derivatives. Homology modeling with a known 3D structure (PDB code: 1K1D) allowed us to identify the amino acids to be mutated at the substrate binding site and in its immediate vicinity to modulate the substrate specificity. Both single and double substituted mutants were generated by site-directed mutagenesis at appropriate sites located inside and outside of the stereochemistry gate loops (SGL) involved in the substrate binding. Substrate specificity and kinetic constant data demonstrate that the replacement of Phe159 and Trp287 with alanine leads to an increase in the enzyme activity towards D,L-5-benzyl and D,L-5-indolylmethyl hydantoins. The length of the side chain and the hydrophobicity of substrates are essential parameters to consider when designing the substrate binding pocket for bulky hydantoins. Our data highlight that D-hydantoinase is the authentic dihydropyrimidinase involved in the pyrimidine reductive catabolic pathway in moderate thermophiles.

## 1. Introduction

Functional, structural and evolutionary relationships of enzymes that cleave the amide ring in cyclic diamide structures have led to the identification of protein families, such as hydrolases, oxidoreductases and amino acid aminotransferases, within a superfamily of amidohydrolytic enzymes [1,2,3]. Optically pure amino acids can be obtained by enantioselective ring opening in 5- and 6-membered hydantoins using cyclic amidohydrolases, known as hydantoinases [1]. It should be recalled that hydantoinase got its conventional name because hydantoin was first obtained by the hydrogenation of allantoin [4]. D-amino acids are naturally tolerant to proteolytic degradation, and hydantoin racemates can be used as promising substrates for the development of semisynthetic antibiotics, peptide hormones, and drugs [5,6,7].

Enzymes of thermophilic bacteria in comparison with their mesophilic counterparts have higher biological and chemical stability, which is a valuable operational criterion for the development of biocatalysts [8,9]. Therefore, thermostable hydantoinases are of particular interest for the production of D-amino acids [10]. D-specific hydantoinases of the genus *Geobacillus* have a high level of sequence similarity (Figure S1) that simplifies comparative enzyme assays and the mutual extrapolation of data [10,11,12]. However, despite 92% sequence similarity, D-hydantoinase of *G. thermocatenulatus* has a broader substrate specificity than the enzyme of *G. stearothermophilus* SD1 [13]. 

The apo-crystal 3D structure of D-hydantoinase from *G. stearothermophilus* SD1 at a resolution of 3.0 Å is a metal-dependent tetrameric protein [14]. The structural features described for D-hydantoinase have been detected in related enzymes from organisms of both prokaryotic and eukaryotic origins [15]. *G. stearothermophilus* SD1 D-hydantoinase represents eight parallel β-sheets linked by eight α-helices, known as a (β/α)_8_-barrel folded structure [14,16]. Three stereochemistry gate loops (SGLs) play major roles in the substrate specificity and enantioselectivity of bacterial D-hydantoinase [14,17]. Homology modeling of *G. stearothermophilus* SD1 D-hydantoinase with a *Thermus sp.* dihydropyrimidinase revealed that both enzymes possess a binuclear metal binding site, partially presented in SGLs, to coordinate Zn^2+^ ion interactions [16,18]. 

Hydrophobic residues, such as Leu65 located at SGL1 (the region from the position His60 to Asp73) and Phe152 and Phe159 at SGL3 (the region from Lys150 to Asp162), have been substituted by smaller or equivalent size amino acids present in D-hydantoinase of *G. thermocatenulatus* [13] and phenylhydantoinase of *Escherichia coli* [19], and the mutant enzymes have been tested to hydrolyze small hydantoin and bulky hydroxyphenylhydantoin [16]. It turns out that the mutations at Leu65 and Phe159 increase the relative activity of D,L-phenyl-hydantoin and D,L-hydroxyphenyl-hydantoin but decrease the activity toward hydantoin compared to the wild type hydantoinase. The single substitution F159A and double substitution L65F/F159A mutants exhibit remarkable increase in relative activity of aromatic substrates, which is associated with a decrease in the activity of hydantoin. Notably, there is a gradual increase in the kinetic constant *k*_cat_ for aromatic substrates associated with the decrease of the amino acid side chain. It has been suggested that the mutations to smaller amino acids at corresponding positions enlarge the substrate binding pocket, which leads to a loose interaction with corresponding substrates [16]. Next, the authors applied a rational design of the optimized size and hydrophobicity of amino acids to be mutated in D-hydantoinase from *G. stearothermophilus* SD1 and selected a double mutant M63I/F159S that showed more than five-fold increase in activity for hydroxyphenyl-hydantoin compared to the wild type enzyme [20]. In this regard, in silico computing of amino acid sequences to design protein folding that corresponds to the desired function appears to be a promising way to improve biocatalysts for industrial processes [21].

The difference in the substrate specificity of hydantoinases and other cyclic amidohydrolases, and the scarcity of data on their roles in metabolic pathways in bacteria emphasizes the necessity for further studies to gain greater insight into their structure–function relationships in evolutionary distant organisms. Herewith, we use a rational redesign of new amino acids to be mutated in the substrate binding pocket of a recombinant D-hydantoinase from *G. stearothermophilus* ATCC31783 to increase the substrate specificity for bulky side chain hydantoins. 

## 2. Materials and Methods

### 2.1. Chemicals

The enantioselective substrates D-5-(2-methylthioethyl) hydantoin, L-5-(2-methylthioethyl) hydantoin, and D,L-5-(2- methylthioethyl) hydantoin were obtained from Rhône-Poulenc Nutrition Animale (Lyon, France). 5-Benzyl hydantoin and 5-indolylmethyl hydantoin were synthesized as previously described [22]. All other hydantoinase substrates were purchased from Sigma Aldrich (Saint-Quentin-Fallavier, France).

### 2.2. Enzyme Assays

*G. stearothermophilus* ATCC 31,783 was grown in LB-broth (pH 7.3) at 56 °C. The *E. coli* BL21 (DE3)/pET-Hyd strain carrying the cloned wild type or mutant hydantoinase gene [10] was grown in LB-broth at 25 °C to reduce the aggregation of the overexpressed enzyme. Bacterial cultures were harvested by centrifugation and sonicated at 19 kHz at 4 °C. The recombinant D-hydantoinase activity was measured in 0.1 M MOPS (pH 8.0) supplemented with 0.5 mM MnSO_4_ and 20–50 mM hydantoin substrates. Short incubation times were probed and chosen to minimize chemical modification of the *N*-hydantoin derivatives under alkaline conditions [23]. The reaction was stopped by adding TCA (4% final concentration) and dimethylaminobenzaldehyde (10% *w*/*v* in 6 M HCl). After centrifugation (5000× *g*, 5 min), the amounts of *N*-carbamoyl derivatives were quantified at 430 nm [6]. For quantitative analysis, the reaction was stopped with phosphoric acid (0.2 M final concentration) and analyzed by HPLC (Kontron, Toulon, France) using a Luna Cl8 column (Phenomenex, Torrance, CA, USA). The mobile phases used were 25 mM phosphate buffer (pH 2.5) at a flow rate of 1 mL/min for the dihydropyrimidinase activity or 0.1 M phosphoric acid and methanol (85–15 *v*/*v*) at a flow rate of 0.9 mL/min for the hydantoinase activity. Detection was carried out at 215 nm. One unit (U) of the activity was defined as the amount of enzyme that catalyzed the hydrolysis of 1 µmol of dihydrouracil or dihydrothymine per minute or as the formation of 1 µmol of *N*-carbamoyl-methionine per minute, under standard conditions. The concentration of protein was measured by the modified Folin phenol method [24]. The specific activity of the enzyme is expressed in U/mg protein. Kinetic parameters and their variances were calculated by a multivariate linear regression [25] using Gauss 4.0 software (Aptech Systems, Inc., Higley, AL, USA) and the program described previously [26]. The Michaelis constant (Km) and maximum velocity of the reaction (Vmax) catalyzed by D-hydantoinase were calculated by measuring the enzyme activity at the range of substrate concentrations of more than one order, considering the values of Km.

### 2.3. Homology Modeling

The homology models of *G. stearothemophilus* ATCC 31,783 wild type hydantoinase and mutant enzymes to be generated were created with the SWISS-MODEL homology-modelling server [27,28]. The best template among the available 3D structure options offered by BLAST search was detected according to the highest identity, 97%. The quality of generated models was verified by ProSa-web [29].

### 2.4. Site-Directed Mutagenesis

PCR amplification primers for mutagenesis (Table S1) were synthesized by Eurofins (Les Ulis, France). Site-directed mutagenesis of the hydantoinase gene from *G. stearothemophilus* ATCC 31,783 was performed by selection of mutations in *E. coli* XL1-Blue cells according to the recommendations of the QuickChange protocol (Agilent, Santa Clara, CA, USA). The coding region of hydantoinase was sequenced in putatively mutated clones and the clones that acquired the designed mutations were used for enzyme assays. DNA sequencing was performed with the 3730 × 1 DNA analyzer by Macrogen (Seoul, Republic of Korea).

### 2.5. Long Inverse PCR

Previously, we used inverse PCR for amplification of DNA regions upstream and downstream of the gene coding for D-hydantoinase in *G. stearothemophilus* ATCC 31,783 [10]. However, no details of the method used were described, but they are now presented below. The oligonucleotide primers F1 and R1 of 33-mer, oriented in inverse directions, were designed to have high melting temperatures for the amplification of long DNA segments (Table S1). DNA gently extracted from *G. stearothermophilus* ATCC 31,783 was digested with *Eco*RI or *Pst*I, precipitated by ethanol, and dissolved in sterile water to a final concentration of 1 ng/µL, and then self-ligated by 2.8 Weiss units of T4 polynucleotide ligase (Takara Shuzo Co., Ltd, Kyoto, Japan) at 16 °C for 30 min. DNA was precipitated with ethanol, dissolved in water and after heating at 94 °C for 10 min, it was amplified with 2.5 units of LA Taq polymerase under the following conditions: denaturation at 98 °C for 5 s or 20 s, combined annealing and elongation at 68 °C for 15 min for 14 cycles, and then under the same conditions but with 30 s extension of the annealing/elongation steps for more than 16 cycles. PCR-amplified DNA and total bacterial DNA were digested with restriction enzymes and hybridized with a hydantoinase-specific DNA labeled by digoxigenin (DIG). The amplified DNA samples were directly used for DNA sequencing.

### 2.6. Molecular Docking

A 3D structure of hydantoin substrates was created by ChemBioDraw Ultra 12.0 software. In silico molecular interactions were modeled with the AutoDock Vina software package [30]. Free energy minimization of substrate molecules was generated by the MM2 program integrated in ChemBioDraw Ultra 12.0. To visualize protein–substrate interactions, the AutoDock Tools 1.5.6 program (San Diego, CA, USA) was used. 

### 2.7. Statistics

The average values of the enzyme activity were estimated from three measurements of independent assays. Calculations were performed with Microsoft Excel. Standard deviations were calculated from three experiments.

## 3. Results

### 3.1. Characterization of G. stearothermophilus Hydantoinase

A significant portion of a 52 kDa hydantoinase of *G. stearothermophilus* ATCC 31,783 was detected as an aggregated protein in E. coli BL21(DE3)/pET-Hyd cells. Two approaches were tested to purify the overexpressed enzyme.

The solubilization of the pellet in 0.1 M MOPS (pH 8.0) containing 0.2% N-lauroyl sarcosine for 24 h as described in [31] resulted in high amounts of soluble and pure hydantoinase (Figure S1A). However, the enzyme was inactive, and our attempts to restore the activity failed when using renaturation protocols. According to the second approach, the culture of the overexpressed D-hydantoinase was sonicated, heat-treated and centrifuged to discard the denatured mesophilic host proteins, as described previously [32]. The supernatant was subjected to ultrafiltration on a 30 kDa column (Amicon) to eliminate small proteins of less than 30 kDa and then desalted on a PD-10 column (Pharmacia). This approach provided a sufficient yield of purified and active protein for use in enzyme assays (Figure S1B). 

The hydantoinase activity was measured in a MOPS-boric acid buffer at different temperatures (Figure S2A) and pH levels (Figure S2B). The enzyme was stable in the temperature range from 45 to 65 °C (Figure S2C) and it showed high activity in the pH range from 8.0 to 9.2 after incubation at 65 °C for 20 min (Figure S2D). The enzyme activity was highest at 0.5 mM manganese (Figure S2E).

The reactions carried out with seven divalent metals in 0.1 M MOPS buffer (pH 8.0) at 60 °C for 30 min showed that Mn^2+^ ions remarkably enhance the hydrolysis of hydantoin compared to six other metals, including Zn^2+^, which have weaker effects on the enzyme (Table 1). Therefore, *G. stearothermophilus* ATCC 31,783 hydantoinase is most likely an Mn^2+^ dependent enzyme that distinguishes it from the Zn^2+^ dependent hydantoinase of *G. stearothermophilus* SD1 [14]. A positive effect of Mn^2+^ was also observed for *Pseudomonas putida* D-hydantoinase expressed in *E. coli* [33].

It was previously shown that cell extracts of *G. stearothermophilus* ATCC 31,783 and ATCC 31,195 hydrolyze D-5-(2-methylthioethyl) hydantoin [10]. A quantitative analysis of the stereospecificity of the purified enzyme confirmed that it exclusively hydrolyzes D-5-(2-methylthioethyl) hydantoin to D-carbamoyl methionine (more than 98%) and is not active with L-5-(2-methylthioethyl) hydantoin (1,6%) compared to the substrate used (Table 2). Therefore, it was concluded that *G. stearothermophilus* hydantoinase is a strict D-stereospecific enzyme.

The specific activity of the recombinant D-hydantoinase was measured in MOPS buffer (pH 8.0) at 60 °C supplemented with substrates of different sizes. The wild type enzyme was found to be active towards dihydrouracil, hydantoin, D,L-5-methylhydantoin, and D,L-5-(2-methyl-thioethyl)-hydantoin, as opposed to D,L-5-benzyl-hydantoin and D,L-5-indolylmethyl hydantoin (Table 3). Its specific activity was 100-fold higher for dihydrouracil, considered as the best substrate, than for bulk D,L-5-benzyl-hydantoin and D,L-5-indolylmethyl hydantoin under the conditions used. These data suggested that the catalytic site in D-hydantoinase is not accessible for bulky substances.

### 3.2. Homology Modeling of Thermostable D-Hydantoinase

To study structure–function features and molecular interactions of the wild-type D-hydantoinase of *G. stearothermophilus* ATCC 31783, we used the homology modelling approach relative to a 3D structure of amidohydrolases at 3 Å and higher resolution. To create a reliable model, a search was performed in databases by checking 50 possible options responding to the criteria of 97–15% identity and a coverage of 0.97–0.7. D-hydantoinase from *G. stearothermophilus* SD1 (PDB code: 1K1D) was identified as the most appropriate template in terms of the overall quality of the model structure with a z-score equal to 9.64 estimated using the interactive ProSa service [29]. 

Docking analysis of the model D-hydantoinase from *G. stearothermophilus* ATCC 31,783 indicated that dihydrouracil, hydantoin, D,L-5-methylhydantoin, and 5-(2-methylthioethyl)-hydantoin have relatively high binding ability compared to 5-benzyl hydantoin and 5-indolylmethyl hydantoin. This suggests that bulky substrates cannot access the binding pocket or that aromatic substrates are nonoptimized structures relative to hydrophobic amino acids in the pocket.

**Figure 1:**
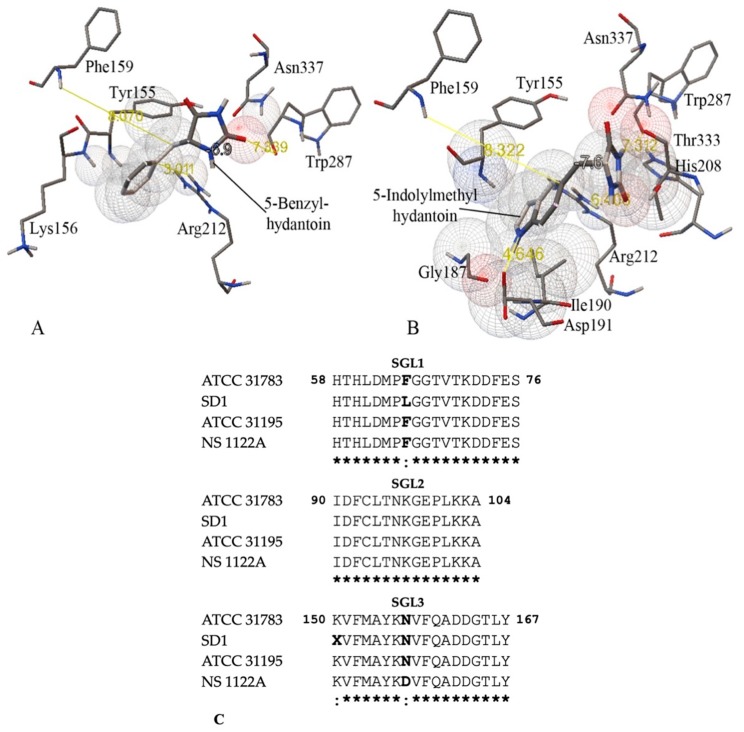
Amino acid interactions with 5-benzyl hydantoin at ΔG −6.9 kcal/mol (**A**) and 5-indolylmethylhydantoin at ΔG −7.6 kcal/mol (**B**) in the substrate binding pocket of a modelled D-hydantoinase from *G. stearothermophilus* ATCC 31783. Amino acids are shown as thin molecules, and the ligands are shown as thick molecules. The electronic clouds for atoms of interacting residues and ligands are presented in the form of spherical grids. The distance (Å) is depicted in yellow. Regions of conserved SGLs (**C**) include *G. stearothermophilus* ATCC 31,783 [10], *G. stearothermophilus* SD1 [12], *G. stearothermophilus* ATCC 31,195 [10], and *G. stearothermophilus* NS1122A [11].

We sought to improve the affinity of D-hydantoinase for bulky substrates by replacing amino acids in the substrate binding pocket or in close proximity to the catalytic site (Figure 1A,B). This goal could be achieved by enlarging the binding space through the mutagenesis of SGLs to accommodate the large hydroxyphenyl ring of D-hydroxyphenyl hydantoin by the substitution of Phe159 in SGL3 (Figure 1C). Moreover, the amino acids Ile190, Arg212, and Trp287, which are all located outside of SGLs but in the immediate vicinity of an aromatic ring or hydantoin ring of aromatic substrates (Figure 1A,B), might also influence the hydrolysis of 5-benzyl hydantoin and 5-indolylmethylhydantoin. In this context, the alignment of 28 hydantoinases from different organisms showed 100% identity of Arg212 and 71% and 57% identity of Ile190 and Trp287, respectively. This conservation of amino acids in protein sequences suggests the structural and/or functional importance of these residues in the enzyme evolution. Therefore, this valuable information was applied as a rational prediction to replace the selected amino acids in the wild type D-hydantoinase of *G. stearothermophilus* ATCC 31,783 and to obtain a mutant enzyme with an improved substrate specificity.

### 3.3. Substrate Specificity and Kinetic Constants of Engineered Mutants of D-Hydantoinase

Next, we used site-directed mutagenesis to replace the amino acids at positions Phe159, Ile190, and Trp287 with a smaller alanine residue to conserve the hydrophobic nature of the mutated sites, and Arg 212 with lysine in D-hydantoinase from *G. stearothermophilus* ATCC 31783. The mutations generated were verified by DNA sequencing of the entire hydantoinase gene, and the approved construct of each mutant enzyme was tested towards different substrates.

The specific activity of the mutants F159A, I190A, R212K, and W287A towards substrates was obviously different from that of the wild type D-hydantoinase, thereby demonstrating that single substitutions of corresponding amino acids affect the activity of the mutant enzyme (Table 3). Amino acid replacement in the mutant W287A, and to a lesser extent in F159A, with alanine remarkably increased the activity of both enzymes towards the bulky substrates 5-benzyl hydantoin and 5-indolylmethylhydantoin and decreased the activity towards other substances, except dihydrouracil, by F159A. Mutations in I190A and R212K decreased the enzyme activity towards four substrates without affecting low levels of the activity with dihydrouracil and 5-(2-methylthioethyl)-hydantoin as substrates.

Inspired by the positive effects of W287A and F159A mutant enzymes in enhancing the hydrolysis efficacy of 5-benzylhydantoin and 5-indolylmethylhydantoin, we designed the double substitution mutants W287A/F159A and W287A/R212K with a hope of improving the accessibility of the substrates to the catalytic pocket. The double mutant W287A/F159A exhibited levels of enzyme activity towards 5-benzylhydantoin and 5-indolylmethylhydantoin similar to the single mutants W287A and F159A, but with a loss of activity for dihydrouracil compared to that of F159A (Table 3). The double mutant W287A/R212K exhibited no reasonable modulation in enzyme activity compared to the single mutation counterpart R212K. All mutant enzymes hydrolyzed D-5-(2-methylthioethyl) hydantoin, proving D-stereospecificity.

To verify the effect of mutations on the substrate affinity, we calculated the Michaelis constant (K_m_), catalytic efficiency (k_cat_/K_m_), and catalytic constant (k_cat_) for dihydrouracil and 5-indolylmethyl hydantoin, as suggested in [34]. The single mutations W287A and F159A have no effect on the catalytic efficiency with dihydrouracil, whereas the double mutations W287A/F59A lead to a 10-fold decrease in the value of k_cat_/K_m_ (Table 4). Regarding the K_m_ constants, the values obtained suggest controversial behavior for single mutants W287A and F159A. This means that the double mutant W287A/F159A displays a drastic decrease in the catalytic efficiency as compared with that of single mutants W287A and F159A. No significant changes were observed for the mutant I190A towards dihydrouracil. Wild type D-hydantoinase showed Km values 2.5-fold and 2.3-fold higher, respectively for D,L-5-methylhydantoin and 5-(2-methylthioethyl)-hydantoin, compared to dihydrouracil. 

Thus, the kinetic constants confirm the results of substrate specificity, i.e., that single substitutions of Phe159 and Trp287 with alanine in D-hydantoinase lead to an increase in the activity towards D,L-5-indolylmethyl hydantoin.

### 3.4. D-Hydantoinase Is the Enzyme of the Reductive Catabolism of Pyrimidines in G. stearothermophilus

The discovery of a gene encoding L-hydantoinase on plasmid DNA in *Pseudomonas* sp. NS671 [35] suggests the extrachromosomal nature of the gene in other bacteria. We detected several plasmids, including 350 kb megaplasmids, in *G. stearothermophilus* ATCC 31,783 and ATCC 31,195 by applying the pulsed-field gel electrophoresis method [36]. However, the D-hydantoinase coding sequence was proven to be present in the chromosome of both strains, suggesting an essential role of the enzyme rather than a transitory function in bacteria. To understand the role of the enzyme providing D-specific chirality in *G. stearothermophilus* better, we sought to study the region surrounding the coding sequence for the D-hydantoinase gene.

**Figure 2:**
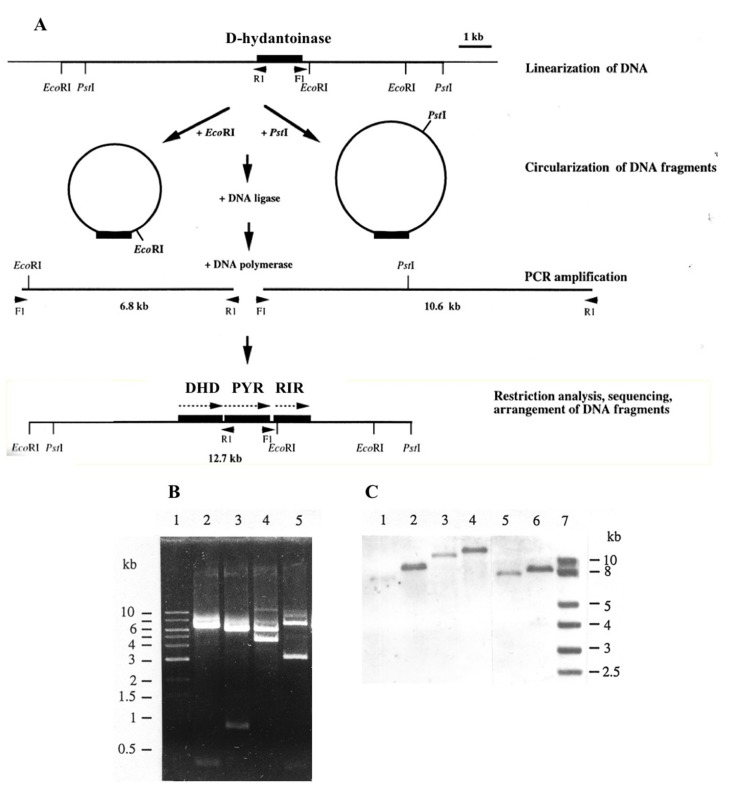
Strategy of amplification of a D-hydantoinase gene cluster from *G. stearothermophilus* ATCC 31,783 by long inverse PCR (**A**), and genetic analysis of synthesized DNA fragments (**B**,**C**). Abbreviations: DHD—dihydropyrimidine dehydrogenase, PYR—dihydropyrimidinase (or D-hydantoinse), RIR—ribonucleotide reductase. Restriction analysis (**B**): Lanes l—molecular markers; 2 and 3—a 6.8 kb DNA fragment digested with *Eco*RI and *Pst*I, respectively; 4 and 5—a 10.6 kb DNA fragment digested with *Pst*I and *Eco*RI, respectively. Southern blot analysis (**C**): PCR-amplified DNA and total bacterial DNA hybridized with DIG-labeled hydantoinase-specific probe. Lanes 1 and 3—non-digested 6.8 kb and 10.6 kb DNA fragments, respectively; 2 and 4—total DNA digested with *Eco*RI and *PstI*, respectively; 5—a 10.6 kb DNA fragment digested with *Eco*RI; 6—total DNA digested with *Eco*RI and *Pst*I; 7—molecular markers.

In the absence of *G. stearothermophilus* genome sequence information, we took the advantage of inverse PCR [37,38] to amplify DNA segments upstream and downstream of the gene coding for D-hydantoinase. The DNA of *G. stearothermophilus* ATCC 31,783 was linearized by the restriction endonuclease EcoRl or Pstl, for which no recognition site was found in the gene, and then treated with DNA ligase (Figure 2A). A putative circularized DNA was used as a template for amplification with long oligonucleotide primers oriented in the inverse directions. Large DNA fragments of 6.8 kb and 10.6 kb were obtained with primary digested EcoRI and Pstl DNA, respectively (Figure 2B). Southern blot hybridization confirmed that the cloned DNA fragments were 1.3 kb shorter, which corresponds to the non-amplified internal region between F1 and R1 primers (Figure 2C). Two overlapping regions of DNA were arranged into one continuous chromosomal region and used directly for DNA sequencing.

**Figure 3:**
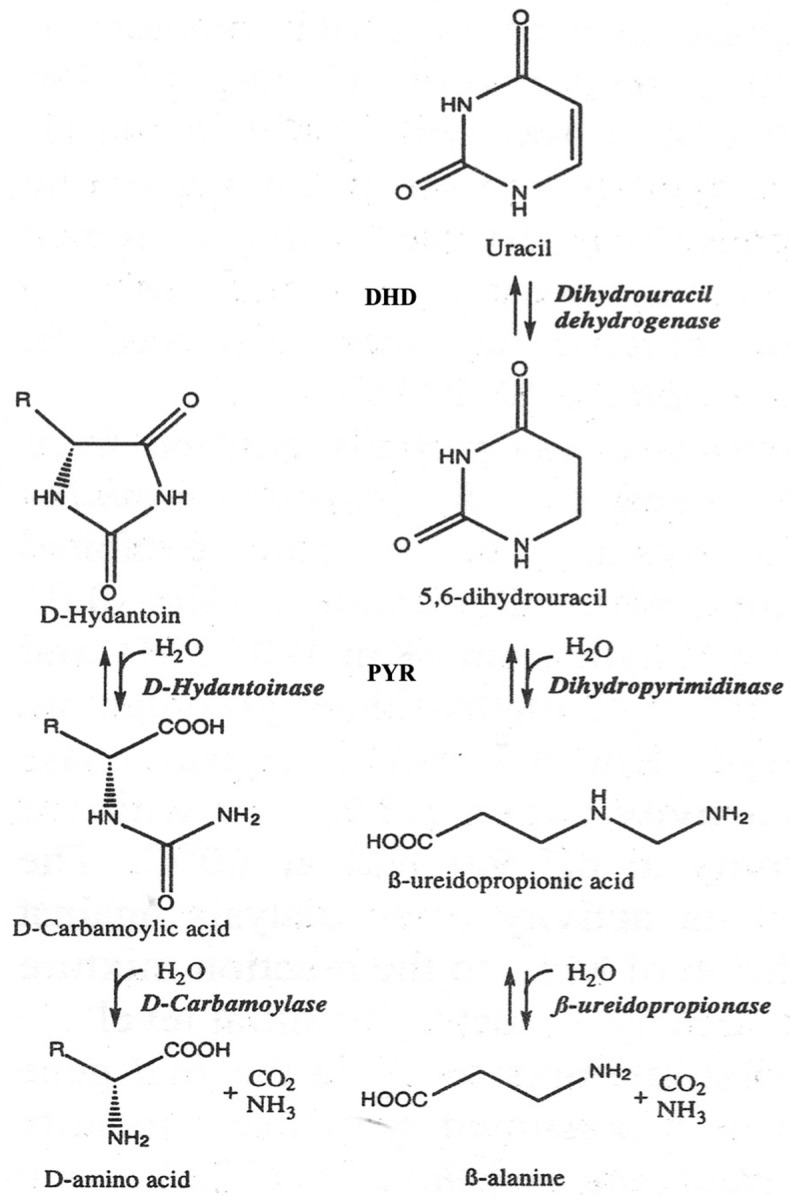
Enantioselective ring opening in D-hydantoin with D-hydantoinase or dihydropyrimidinase (PYR) and reductive pyrimidine catabolic pathway, in which dihydropyrimidine dehydrogenase (DHD) and dihydropyrimidinase catalyze the first two reactions in *G. stearothermophilus*.

The comparison of the sequenced DNA with genomic bank data allowed us to identify a cluster of three genes encoding dihydropyrimidine dehydrogenase, dihydropyrimidinase, and ribonucleotide reductase, all oriented in the same direction in the genome of *G. stearothermophilus* ATCC 31,783 (Figure 2). Crucially, the sequence coding for D-hydantoinase corresponds to the gene encoding dihydropyrimidinase, which is involved in the reductive catabolism pathway of pyrimidines (Figure 3). 

## 4. Discussion

The amidohydrolase superfamily is characterized by (β/α)8-barrel fold amidohydrolases and the presence of mononuclear or binuclear metal binding centers for one or two metal ions [39]. Two metal ions are bridged by a carboxylated amino acid, usually a lysine residue, to coordinate the metal binding in the binuclear metal binding site (Figure S3). Mononuclear and binuclear centers can be occupied by zinc ions, which interact with four histidine residues and one aspartic acid residue, which are highly conserved in the N-terminal and C-terminal regions of the proteins [40]. Notably, replacements of amino acid residues involved in the metal binding with alanine abolish D-hydantoinase activity [41], and the pH profiles of a metal reconstituted enzyme depend on a particular metal ion bound to the substrate binding site [42].

D-hydantoinase of *G. stearothermophilus* SD1 has been shown to be a binuclear metal-binding enzyme and Zn^2+^ ions interact with amino acids at positions His58 and His60 in SGL1, His183, His239, Asp 390, and probably Lys150 in SGL3 (Figure 1, Figure S4) [14,16]. Our data revealed that the recombinant D-hydantoinase from *G. stearothermophilus* ATCC 31,783 is remarkably activated by Mn^2+^ ions, whereas Zn^2+^ and other bivalent metals have weaker effects on the enzyme activity (Table 2). Obviously, this feature contrasts with the Zn^2+^ dependence of the enzyme of *G. stearothermophilus* SD1 considering a 97.4% sequence identity of proteins [36]. However, the sequence comparison reveals that two proteins differ in terms of the amino acids located near sites involved in the metal binding, namely, Phe65 versus Leu65 in SGL1 and Phe149 versus Leu149 in *G. stearothermophilus* ATCC 31,783 and *G. stearothermophilus* SD1, respectively (Figure 1, Figure S4). According to the reaction mechanism, metal ions deprotonate a water molecule to conduct a nucleophilic attack on the substrate in the active site of amidohydrolases [39]. Therefore, the presence of Phe65 instead of Leu65 close to His58 and His60, all located in SGL1 and participating in the binding to substrates, may affect the choice of metal to interact with the enzyme. In addition, aromatic Phe149 instead of Leu149, located ahead of the catalytic Lys150, can prevent or retard the carboxylation of lysine and differentiate between the bivalent metals Zn^2+^ and Mn^2+^ for bridge formation in the enzyme. This logical assumption is also justified by the fact that the 3D structure of *G. stearothermophilus* SD1 hydantoinase has been resolved with the apo-protein, in the absence of metal ions [14].

A large number of enzymes are activated by manganese that serves as a cofactor for the reactions catalyzed by metalloenzymes [43]. In this context, bacterial hydantoinase and allantoinase, which are involved in pyrimidine or purine degradation in bacteria, possess manganese dependence rather than zinc dependence [33,44]. Moreover, recent data show that the presence of Mn^2+^ in the metal binding site in cyclic amidohydrolases is a more frequent event than thought previously [45]. Manganese plays an important role in different enzyme-catalyzed reactions, including hydrolysis, because of its ability to readily change oxidation state [46]. However, Mn^2+^ exhibits fast ligand exchange kinetics and can easily be replaced by other divalent metal ions, such as Zn^2+^. Therefore, the revelation that magnesium is the essential metal required for the catalytic activity of D-hydantonase is important for the formulation of optimal conditions for reactions carried out with biocatalysts.

The structural architecture of the substrate binding and metal binding sites is highly conserved in cyclic amidohydrolases of different origins, including D-hydantoinases (Figure S3, Figure S4). Assuming the significance of amino acids located in the active site or in its proximity in providing the substrate specificity according to homology modeling, we sought to enlarge the substrate binding pocket for bulky substrates in D-hydantoinase of *G. stearothermophilus* ATCC 31783. Single and double mutations were generated in a SGL3 and outside of SGLs in the immediate vicinity of the active site. The single replacement of Phe159 by alanine remarkably increased the specific activity of the mutant D-hydantoinase in accordance with the data described for *G. stearothermophilus* SD-1 [16,19].

It is noteworthy that our search revealed mutations that have not yet been described outside of SGL, which remarkably enhance the activity of the enzyme. Replacing the tryptophan residue with alanine allowed us to create a mutant enzyme W287A with approximately 40-fold higher specific activity for bulky D,L-5-benzyl hydantoin and D,L-5-indolylmethyl hydantoin compared to the wild type enzyme, which is deprived of activity towards these substrates (Table 3). On the other hand, the replacement of Arg212 by lysine in the mutant R212K decreases the enzyme activity for small substrates without modulating the activity towards bulky substrates. 

Our data show that the hydrophobicity in the proximity of the enlarged pocket in mutant D-hydantoinase is likely important to facilitate the recognition of bulky substrates or the binding to the active site. However, coordination of the optimal size of substrates in the enlarged hydrophobic pocket is also important to maintain the enzyme’s functionality for smaller substrates during engineering of the enzyme with a broader substrate specificity.

Studies of the substrate specificity of hydantoinases indicate their relationship with dihydropyrimidinases [1], which has been affirmed by the similarity of 3D structures [16,47]. We showed that native D-hydantoinases of *G. stearothermophilus* ATCC 31,783 and ATCC 31,195 and their recombinant versions expressed in *E. coli* exhibit high activity towards dihydrothymine and dihydrouracil and not for dihydroorotate [36]. Given these results and the high levels of protein similarity, one can definitively conclude that D-hydantoinase is in fact dihydropyrimidinase, an authentic enzyme of *G. stearothermophilus*. The close location of three genes identified as encoding for dihydropyrimidine dehydrogenase, dihydropyrimidinase (D-hydantoinase), and ribonucleotide reductase, and transcribed in the same direction (Figure 2), strongly suggests their organization in an operon, providing catalytic functions for the same pathway in bacteria. Hence, the “hydantoin operon” is involved in the pyrimidine reductive catabolic pathway, which is important for the utilization of uracil and thymine as sources of nitrogen and carbon in bacteria of the genus *Geobacillus*. In this pathway, pyrimidine deoxyribonucleosides are first reduced by dihydropyrimidine dehydrogenase with NADPH as a cofactor to 5,6-dihydrouracil and 5,6-dihydrothymine, which then are cleaved by dihydropyrimidinase (Figure 3). The third enzyme, ribonucleotide reductase, catalyzes the conversion of ribonucleotides to deoxyribonucleotides, which can be used not only as the building blocks for DNA replication [48] but also as the substrates for reductive degradation through the “hydantoin operon” in thermophiles. Notably, unbalanced deoxyribonucleotide levels lead to the increase in the mutation rate [49], which emphasizes the essential roles of dihydropyrimidine dehydrogenase and dihydropyrimidinase (D-hydantoinase) in maintaining the balance of DNA precursors in cells. 

## 5. Conclusions

D-hydantoinase (dihydropyrimidinase) of *G. stearohermophilus* provides stereospecific cleavage of 5- and 6-membered cyclic diamide structures. The manganese-dependent enzyme has limited substrate specificity for small hydantoins that can be explained by the importance of its authentic function in the metabolism of moderate thermophiles. Virtual analysis has suggested a rational way to enlarge the substrate binding pocket for large hydantoins. This prediction has been experimentally proven by engineering mutant proteins in which the target amino acids are substituted inside and outside the catalytic site, or both. Promising mutant enzymes have been selected that are capable of cleaving bulky hydantoin derivatives. The role of dihydropyrimidinase in the processes associated with the reductive catabolism of pyrimidines should be considered for further diversification and improvement of the performance of biocatalysts, in particular in whole-cell systems of host bacteria.

In this study, we have designed the long inverse PCR to amplify large DNA fragments around the desired gene. This technique, an alternative to genome sequencing, provides valuable sequence information on large regions of bacterial genomes for metagenomic and metaproteomic comparisons.

## Figures and Tables

**Table 1:** Effects of divalent metal ions on specific activity of the recombinant D-hydantoinase from *G. stearothermophilus* ATCC 31783. The activity without metal ions was 0.35 U/mg. ND—not detected.

Divalent Metal Ion	Specific Enzyme Activity, U/mg	
	0.2 mM	2 mM
Mn^2+^	8.37	4.49
Fe^2+^	1.76	1.76
Co^2+^	1.76	ND
Ni^2+^	0.66	ND
Mg^2+^	0.61	1.26
Cu^2+^	0.55	1.56
Zn^2+^	0.55	ND
EDTA	0.35	0.35

**Table 2:** Stereospecificity of the recombinant hydantoinase from *G. stearothermophilus* ATCC 31783. The substrates are at the concentration of 100 mM.

Substrate	D-Carbamoyl Methionine, mM
D, L-5-(2-methylthioethyl) hydantoin	48.9 ± 2.1
D-5-2-methylthioethyl) hydantoin	98.3 ± 2.5
L-5-(2-methylthioethyl) hydantoin	1.6 ± 0.5

**Table 3:** The substrate specificity of the wild type and mutant D-hydantoinase of *G. stearothermophilus* ATCC 31,783 purified from *E. coli* cells. The specific activity, U/mg, was estimated from three independent experiments after subtraction of the activity in the absence of the enzyme sample. The wild type enzyme was also tested to hydrolyze dihydroorotate (no activity detected), allantoin (3.6 U/mg), and dihydrothymine (2.6 U/mg). Standard deviations (±) were estimated from three experiments.

Mutation	Hydantoin	Dihydrouracil	D,L-5-methyl-lhydantoin	D,L-5-(2-methyl-thioethyl)-hydantoin	D,L-5-benzyl-hydantoin	D,L-5-indolylmethyl Hydantoin
Wild type	3.5 ± 0.30	4.0 ± 0.31	1.4 ± 0.10	1.2 ± 0.09	< 0.04	< 0.04
W287A	0.05 ± 0.01	0.43 ± 0.03	0.17 ± 0.02	0.54 ± 0.04	1.75 ± 0.12	1.57 ± 0.12
F159A	0.06 ± 0.01	5.50 ± 0.40	< 0.04	0.58 ± 0.04	1.30 ± 0.10	1.10 ± 0.08
W287A/F159A	0.32 ± 0.02	0.85 ± 0.07	0.53 ± 0.03	0.08 ± 0.01	1.75 ± 0.15	1.16 ± 0.09
I190A	0.56 ± 0.04	2.2 ± 0.10	0.22 ± 0.01	0.53 ± 0.04	0.05 ± 0.01	< 0.04
R212K	1.19 ± 0.10	0.06 ± 0.01	0.22 ± 0.01	0.5 ± 0.04	< 0.04	< 0.04
W287A/R212K	1.1 ± 0.10	< 0.04	< 0.04	0.27 ± 0.02	0.5 ± 0.04	< 0.04

**Table 4:** Kinetic constants estimated for wild type and mutant D-hydantoinases. Due to a very low activity of the enzyme for D,L-5-indolylmethyl hydantoin, K_m_ and kcat could not be evaluated for the wild type and I190A mutant enzymes.

Enzyme	Km, mM		kcat/Km, (s·mM) ^−1^		kcat, s^−1^	
	Dihydrouracil	D,L-5-indolylmethyl Hydantoin	Dihydrouracil	D,L-5-indolyl-methyl Hydantoin	Dihydrouracil	D,L-5-indolyl-methyl hydantoin
Wild	0.97 ± 0.22	ND	11.3 ± 2.20	ND	11.2 ± 0.70	ND
W287A	0.43 ± 0.13	1.66 ± 0.85	6.53 ± 1.83	4.13 ± 1.63	2.76 ± 0.17	6.83 ± 0.97
F159A	2.89 ± 1.27	0.60 ± 0.58	7.53 ± 2.50	6.89 ± 5.83	21.8 ± 2.60	4.16 ± 0.60
W287A/F159A	5.61 ± 2.32	4.30 ± 0.40	1.10 ± 0.30	2.90 ± 0.17	6.16 ± 1.00	12.5 ± 0.50
I190A	1.12 ± 0.61	ND	7.09 ± 3.20	ND	7.99 ± 0.90	ND

## Supplementary Materials

The following are available online at https://www.mdpi.com/2571-5135/9/1/5/s1, Table S1. The oligonucleotides of the forward (F) and reverse (R) primers used for PCR amplification. Figure S1. Purification of D-hydantoinase by solubilization with 0.2% N-lauroyl sarcosine (A) and by heat-treatment and ultrafiltration (B). A: Lane 1—crude extract of noninduced cells; 2—crude extract of IPTG-induced cells; 3, 4, 5—washing steps during solubilization of the pellet; 6—hydantoinase in the supernatant after solubilization with N-lauroyl sarcosine; 7—residual proteins in the pellet after solubilization with N-lauroyl sarcosine. B: Lane l—crude extract of IPTG-induced cells; 2—hydantoinase after heat-treatment and ultrafiltration; M—molecular markers, kDa: 250, 150, 100, 75, 50, 37, 25, 20, 15. Figure S2. Effect of temperature (A), pH (B), and manganese (E) on the activity of D-hydantoinase and the stability of the enzyme at different temperatures (C), and different pH levels (D). The error bars represent standard deviations of three experiments. A partially purified enzyme (the specific activity 0.35 U/mg) was incubated in 0.1 M MOPS-Boric acid buffer (pH 8.8), 0.5 mM Mn^2+^, and 50 mM hydantoin for 30 min at different temperatures (A) and in 50 mM MOPS-Boric acid buffer, 0.5 mM Mn^2+^, and 25 mM hydantoin at different pH levels and at 60 °C for 30 min (B). The thermostability of the enzyme was assessed after 30-min pre-incubation at different temperatures (C). The pH- dependence of the enzyme thermostability was assessed after 20-min pre-incubation at different pH levels at 65 °C (D). The influence of Mn^2+^ concentrations was studied in 0.1 M MOPS-Boric acid buffer (pH 8.8) with 50 mM hydantoin at 60 °C for 30 min (E). Figure S3. Alignment of cyclic amidohydrolase sequences. Metal-interacting amino acids are shown in yellow. Figure S4. D-hydantoinases of G. stearothermophilus strains have high levels of sequence similarity. Metal-interacting amino acids are shown in bold and yellow.

## GenBank Accession Number

The accession number of *G. stearothermophilus* D-hydantoinase (dihydropyrimidinase) is MT002440.
