# Supplementary material for: Rational Engineering of the Substrate Specificity of a Thermostable D-Hydantoinase (Dihydropyrimidinase)
Source: High Throughput. 2020 Feb 12;9(1):5. doi: 10.3390/ht9010005 (PMC7175128; doi:10.3390/ht9010005)
Supplement: Supplementary file 1 [file high-throughput-09-00005-s001.pdf]

Rational engineering of the substrate specificity of a thermostable D-hydantoinase (dihydropyrimidinase)

Hovsep Aganyants[1], Pierre Weigel[2], Yeranuhi Hovhannisyan[1], Michèle Lecocq[3], Haykanush Koloyan[1], Artur Hambardzumyan[1], Anichka Hovsepyan[1], Jean-Noël Hallet[2], Vehary Sakanyan[3,4,5*]

Table S1. The oligonucleotides of the forward (F) and reverse (R) primers used for PCR amplification.

Destination	Primer sequences
Trp287Ala	F: 5'-GAAGGCGCGAAATATGTTGCGTCTCCTCCGCTTC<br>R: 5'-GAAGCGGAGGAGACGCAACATATTTCGCGCCTTC
Phe159Ala	F: 5'-CGTATAAAAACGTAGCTCAGGCAGATGATGGAACG<br>R: 5′-CGTTCCATCATCTGCCTGAGCTACGTTTTTATACG
Ile190Ala	F: 5'-CATGCGGAAAATGGTGATGTGGCTGATTATTTAACGAAG<br>R: 5'-CTTCGTTAAATAATCAGCCACATCACCATTTTCCGCATG
Arg212Lys	F: 5'-CATGCATTAACAAAACCTCCAGAATTGGAAGGAG<br>R: 5'-CTCCTTCCAATTCTGGAGGTTTTGTTAATGCATG
Inverse long PCR	F1: 5′-CGGAACCTCGACTATTTCCAAACAGAGCGAGGA<br>R1: 5′-ATCAGCAAGTCCGCTTCATACGTATCCGTTGCG

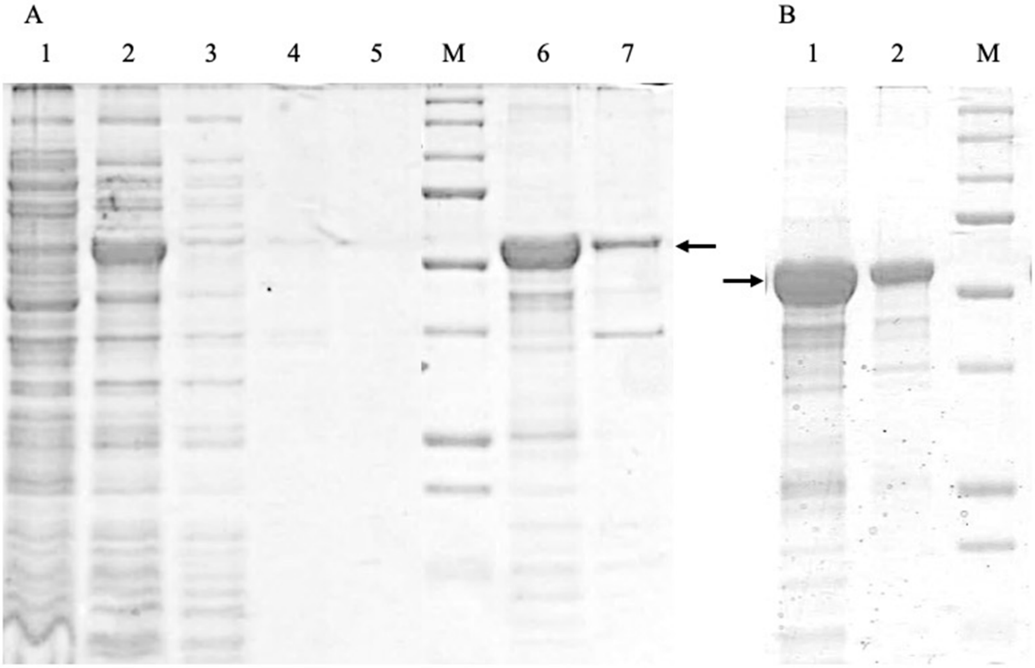

Figure S1. Purification of D-hydantoinase by solubilization with 0.2% N-lauroyl sarcosine (A) and by heat-treatment and ultrafiltration (B). A: Lane 1—crude extract of noninduced cells; 2—crude extract of IPTG-induced cells; 3, 4, 5—washing steps during solubilization of the pellet; 6—hydantoinase in the supernatant after solubilization with N-lauroyl sarcosine; 7—residual proteins in the pellet after solubilization with N-lauroyl sarcosine. B: Lane 1—crude extract of IPTG-induced cells; 2—hydantoinase after heat-treatment and ultrafiltration; M—molecular markers, kDa: 250, 150, 100, 75, 50, 37, 25, 20, 15.

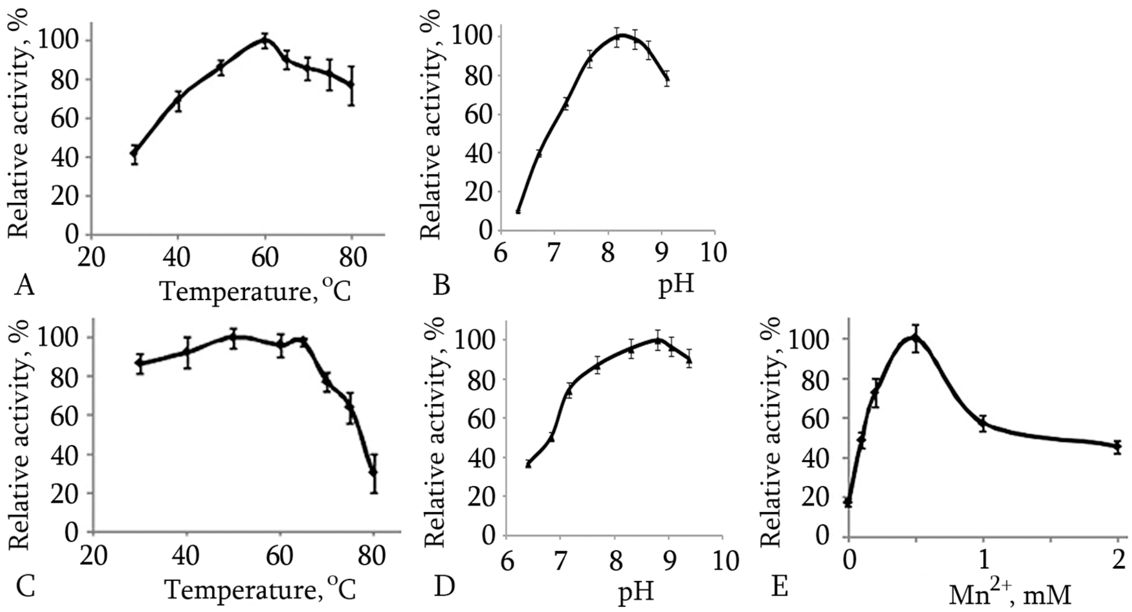

Figure S2. Effect of temperature (A), pH (B), and manganese (E) on the activity of D-hydantoinase and the stability of the enzyme at different temperatures (C), and different pH levels (D). The error bars represent standard deviations of three experiments. A partially purified enzyme (the specific activity 0.35 U/mg) was incubated in 0.1 M MOPS-Boric acid buffer (pH 8.8), 0.5 mM Mn^{2+}, and 50 mM hydantoin for 30 min at different temperatures (A) and in 50 mM MOPS-Boric acid buffer, 0.5 mM Mn^{2+}, and 25 mM hydantoin at different pH levels and at 60 °C for 30 min (B). The thermostability of the enzyme was assessed after 30-min pre-incubation at different temperatures (C). The pH- dependence of the enzyme thermostability was assessed after 20-min pre-incubation at different pH levels at 65 °C (D). The influence of Mn^{2+} concentrations was studied in 0.1 M MOPS-Boric acid buffer (pH 8.8) with 50 mM hydantoin at 60 °C for 30 min (E).

```
                                                                                 # #
1K1D_A        3 KIIKNGTIVTA--------TDTYEAHLLIKDGKIAMIGQNLEekg--aeVIDAKGCYVFPGGIDPHTHLDmplgg----t  68 Geobacillus stea...
NP_769973     8 LIIRGGRVATT--------TDVFEADVAISGETIAAVGRGLPaa---krEIDARGKLVLPGGVDSHAHIEqlsaa---gi  73 Bradyrhizobium j...
AAF69237      6 LIIKNGIICTA--------SDIYAAEIAVNNGKVQLIAASIDpsl-gseVIDAEGAFITPGGIDAHVHVDeplkl---lg  73 Saccharomyces kl...
ZP_00098583   4 LIIKNGTVVSP--------SSSTICDVAIKDDKIVGLGFYEAaeg--irTIDATGKYVMPGVIEAHMHCMapfqg----c  69 Desulfitobacteri...
NP_376926     1 MIFKNARVITP--------KGIIETDFEVEEGKIKKIKKDIVg-----eGKDLSGYYVLPSVIDGHTHFNsrylgakeii  67 Sulfolobus tokodaii
ZP_00005215   5 TVIHGGTIVTP--------TESWQGDLGLVGGRIAGLAERLPgg---arRIDATGRLVLPGGIEAHAHIAqesss---gl  70 Rhodobacter spha...
NP_377042     5 LILKNVKAFTV--------SGPFEGDIAIKDGKIAKIGGDIQeqa--nkVIDLTGKYVVPGLIDGHTHMEfpfmk----e  70 Sulfolobus tokodaii
EAA67040      6 LIITNATIVQPlslpatasDILPNTDIAISGGKIYLLGQNLSslfptapTLSADGAYVLPGGVDSHVHLQqdns------  79 Aspergillus nidu...
ZP_00028774  73 TVVRNARVVTA--------ADTFTSDIGIRDGRIVALGLTLDag---vrEIDAAGRHVTPGGVDSHVHFDqptgd---gs 138 Burkholderia fun...
NP_769935    23 LAIRGGTIVTA--------SDEFRADIGIRDGRIVSIADHLEga---arEIDATGLLALPGGIDSHVHISqasgp---dv  88 Bradyrhizobium j...

1K1D_A       69 vtkDDFESGTIAAAFGGTTTIIDFCLtnk----gepLKKAIETWHNKAngk-avIDYGFHLMISEitd-dvLEELPKVle 142 Geobacillus stea...
NP_769973    74 mnaDTFESATVSAAFGGTTTVIPFAAqhv----gmkLPQVVEDYHALAkkg-avIDYAFHMIIADatretvEEHIPALvk 148 Bradyrhizobium j...
AAF69237     74 dvvDTMEHATRSAVAGGTTTVVAFSTqdvskkgpsaLAESVKLDVDEYseqtlyCDYGLHLILFQie--kpSVEARELld 151 Saccharomyces kl...
ZP_00098583  70 lgaNTFYQQSVSGAFGGVTMFMDFANvfp----gksVLEAVKERRAEMee--saIDFSVHGKFVKspp-elVEEIPQLae 142 Desulfitobacteri...
NP_376926    68 ptaDDYKSGSEITLAGGITSIINFIDpln-----reVTEAVKDEIEKAkst-agIDYSFHLIIKRkd---qINYLPEIik 138 Sulfolobus tokodaii
ZP_00005215  71 msaDDYYTGSVSAAFGGNSSFIPFAAqhr----gqsVDAVIETYDSRAapn-svLDYSYHLIISDptetvlTEELPRAfa 145 Rhodobacter spha...
NP_377042    71 vtaDDFYYGTRAAVAGGVTTIVDFITpak----gqdLLSAYEQWRSNAdpk-viSDYGLHMIIREsnt-kiLEQIPEIin 144 Sulfolobus tokodaii
EAA67040     80 ptgDTWETGTRSAIAGGTTTVLAFASqkrt---dgsLFPVVEEYHRRAsgn-afCDYGFHLILSNptekilAEELPVLvk 155 Aspergillus nidu...
ZP_00028774 139 imaDDFLSGTTSAACGGTTTVIPFACqqr----ghtLREAIDDYHRRAggk-plIDYAFHLIITDptpqvlKEELPTLia 213 Burkholderia fun...
NP_769935    89 vmaDDFASATRAAAAGGNTMVLPFALqek----gtsLRTCVENYRKLAege-cyIDTAFHLIISDptavvlGQELPALvk 163 Bradyrhizobium j...

                               #                                 #
1K1D_A      143 --------eeGITSLXVFMAYKnvfq-addgTLYCTLLAAKELGALVMVHAENGDVIDYLTKKAladgNTDPIYHALTRP 213 Geobacillus stea...
NP_769973   149 ---------qGHASIKIFMTYDrlkv--ddePLLDILLAARQSGAMLCAHAENHGIIAWMVKRLlargYTLPKYHAVSHA 217 Bradyrhizobium j...
AAF69237    152 vqlqaayndyGVSSVKMFMTYPglqi--sdyDIMSAMYATRKNGFTTMLHAENGDMVKWMIEALeeqgLTDAYYHGVSRP 229 Saccharomyces kl...
ZP_00098583 143 ---------yGVPTFKMFMTYKkegvmideeTMLKVFEKAKEVGGLPMLHCEDNTMAEDAIEKVkkigDLSWVNFAKTKP 213 Desulfitobacteri...
NP_376926   139 ---------mGIKSIKMFMAYKgsmq-vddeTIYLVMKKAKELGVTVAIHAENGDVIEVLHNEYk--dKKDAIYHALTRP 206 Sulfolobus tokodaii
ZP_00005215 146 ---------rGITSFKVFMTYDlmnl--gdrGMLDILTVARRHGALTMVHAENNDMVKWMNARLaaagLTAPKYHAISRP 214 Rhodobacter spha...
NP_377042   145 ---------kGVVSFKLFMAYKnefm-lsdgELYKLIKRINDFGGVIGIHAENGEIINELIQQFvsegKIEPIYHYYSRP 214 Sulfolobus tokodaii
EAA67040    156 --------eeGISSVKLYMTYQpmrl--rdsELLDVMGTTRSLGMTTMIHAENADMIDWMTKRLesqgRTEPYAHALARP 225 Aspergillus nidu...
ZP_00028774 214 ---------eGYTSFKIYMTYDalkl--sdrEMLDTLSVARNEGAMVMVHAENADCIAWLTERLleagHTAPRYHATSRP 282 Burkholderia fun...
NP_769935   164 ---------dGYTSFKVFMTYDdlvl--sdkQLLEVFDVARREEALVMVHCEGYDAIRFLTTKLeregHIAPYYHGVSRP 232 Bradyrhizobium j...

                                           #
1K1D_A      214 PELEGEATGRACQLTel--agSQLYVVHVTCAQAVEKIAEARNKGLDVWGETCPQYLVLDqsyle--------------- 276 Geobacillus stea...
```

```
NP_769973     218 RVSEAEAFTRLIGMAal--idQPIMIFHVSTAEGAKVIRDSRGQGLKVFAETCPQYLFLTaadld--------------- 280 Bradyrhizobium j...
AAF69237      230 SIVEGEATNRAITLAtt--mdTPILFVHVSSPQAAEVIKQAQTKGLKVYAETCPQYALLSdaitrchhhgevesygvgid 307 Saccharomyces kl...
ZP_00098583   214 QKCEAAAFERACRLAey--vdCPVMVVHTTHKEALDVARRAHESGFPIYVETGPHYLTLFddny---------------- 275 Desulfitobacteri...
NP_376926     207 VEVEEEAVNRASMLAyl--tgAKTYIVHISSPTSLDIISYWRKKGAKIFSETCPHYLLFDdsyy---------------- 268 Sulfolobus tokodaii
ZP_00005215   215 ALAEAEAINRAISLArl--vgAGLFIVHVSTPEGADLVARAQASGLPIHAETCPQYLAFTrddld--------------- 277 Rhodobacter spha...
NP_377042     215 DIMEIEATNRIASIAsmlgkdVKMYIVHTSTGEAVDIMSSYRKQGFKFYNETVPHYLTLNtdfl---------------- 278 Sulfolobus tokodaii
EAA67040      226 NIAEDEATYRALSLAel--adVPILIVHMSSSVAAKHVRRAQTKLLPVHAETCPHYLFFTseklk--------------- 288 Aspergillus nidu...
ZP_00028774   283 MLVEREATHRAIAFAel--idVPILIVHVSGREAVEQIRWAQSHGLKVYGETCPQYLFLTadslg--------------- 345 Burkholderia fun...
NP_769935     233 QAVEREATHRAISHAei--vgVPIMIVHVSGREAMEQVRWAQQRGLPVHAETCPQYITLTaddmkg-------------- 296 Bradyrhizobium j...

                                                                 #
1K1D_A        277 ------------kpnfEGAKYVWSPPLRek-whQEVLWNALKngqLQTLGSDQCSFDf-------------------kG 323 Geobacillus stea...
NP_769973     281 ------------kpgaDGAKWMCSPPPRth-adQEALWQALSlgdLQTISSDHAPYRfd-------------------eT 328 Bradyrhizobium j...
AAF69237      308 lssisespftnpddrfIGSKYICSPPIRpe-gtQKSIWKGMNngtFTIVGSDHCSYNyyek--------------tstA 371 Saccharomyces kl...
ZP_00098583   276 -------------ekeEGYLYLCSPPLRtp-qdAEDLWQGLQdgtISVTGSDDCTFDtnekaa---------flekdeN 331 Desulfitobacteri...
NP_376926     269 -------------lrsDGNRFIMSPPLRrk-elREELVRKLHm--VNTLGSDYSGFMs--------------------V 311 Sulfolobus tokodaii
ZP_00005215   278 ------------rpgmEGAKYICSPPLRda-atQAALWNHARrgtFESVSSDHAPYRf------------------dA 324 Rhodobacter spha...
NP_377042     279 -------------krpDGYRYVMSPPLRsd-eqRTKLWMRLAsgdIFTVGSDHCVYSd-------------------A 323 Sulfolobus tokodaii
EAA67040      289 ------------gedfRGAMCVCSPALRespmdLKAMWDGLVngtFTTFSSDHAPSKyiflpsllhqlptvaptrfdhqL 356 Aspergillus nidu...
ZP_00028774   346 -----------cddsfEGAKCICSPPPRdk-anQQVIWDGLEngtFEVFSSDHAPFRyd-----------------gpD 395 Burkholderia fun...
NP_769935     297 -----------lnmdiTGAKYVCSPPPRda-esQQAIWEGITsgvFQTFSSDHCPFRyd-----------------dpK 346 Bradyrhizobium j...

1K1D_A        324 QKELGr-------gDFTKIPNGGPIIEDRVSILFSEGVKkGRIT-LNQFVDIVSTRIAKLFglFPKKGTIv-vGSDADLV 394 Geobacillus stea...
NP_769973     329 GKLRAgp-----npNFKQVANGLPGLELRLPLLFDAMVSkGRLG-LEKFVELTATAPAKIYnlHPRKGSIv-vGADADIA 401 Bradyrhizobium j...
AAF69237      372 SKHRAfdpennkngEFRYIPNGLPGVCTRMPLLYDYGYLrGNLTsMMKLVEIQCTNPAKVYgmYPQKGSIlpgVSDADLV 451 Saccharomyces kl...
ZP_00098583   332 GKYIQ---------DFTKVVNGMSGLEVRLPILLSEGAGkGRLT-INQVCALTSTNVAKIYgcYPQKGIIa-pGSDADLV 400 Desulfitobacteri...
NP_376926     312 YKDKAl--------SYIEVPNGVSSTEFLVPTIMSFLFD-NLIT-PEKVAEITSYNQIKLYn-LKEKGFDe--GKDADFT 378 Sulfolobus tokodaii
ZP_00005215   325 SGKFAnga----epAYPAIANGLPGIAMRLPYLFSEGVAaGRIS-LQQFAALSSSNAARLFg-MERKGALl-pGYDADIA 397 Rhodobacter spha...
NP_377042     324 QKKRYree----vpPFHEIPNGVPGTETILPILFYYGVKkGIIG-MERFIEVTSYNPARLFglYPRKGTIm-pGSDADFA 397 Sulfolobus tokodaii
EAA67040      357 GKKKGt-------sSFTQIPNGLPGLETRMPSLFCAGVLtGRLS-VQKFVELTASNPAKLYglSDRKGTIa-pGYDADLV 427 Aspergillus nidu...
ZP_00028774   396 GKRVHgd-----nvSFDKISNGIPGVETRMALLWSEGVRtGRIT-AQSFVALTSTNAAKLYglYPRKGSIa-iGADADLV 468 Burkholderia fun...
NP_769935     347 GKLTPns-----rtSFRWVPNGIPGVETRLPILFSEGVSkGRIS-LQKFVELTATNHARIYglYPRKGSIg-vGFDADIV 419 Bradyrhizobium j...

1K1D_A        395 IFDPNIERVISAethh-----------------mavDYNAFEGMKVTGEPVSVLCRGEFVVRd--KQFVGKPGYG 450 Geobacillus stearothe...
NP_769973     402 IWDPNRETVIADemmh-----------------dlaGYTPFAGRKVKGWPVSVLSRGRVIVEg--NKCLASAGSG 457 Bradyrhizobium japoni...
AAF69237      452 IWYPDDSKKEYNskpklitn---------klmehncDYTPFEGIEIKNWPRYTIVKGKIVYKe--GEILKENADG 515 Saccharomyces kluyveri
ZP_00098583   401 IVDMAQEVTLSKdilh-----------------nniSYCLHEGFKVKGYPIMTIARGKVIVEn--GEFRGEKGAG 456 Desulfitobacterium ha...
NP_376926     379 VIKREEWIVKDWhg-------------------kmDYSIYEGVKFKAKVIQTYLRGELTFD---EDYKGSRGKL 430 Sulfolobus tokodaii
```

```
ZP_00005215 398 IWNPEETREVSLadqh-----------------damDYTPFEGMRLTGWPEHVLSRGETVVEa--GELKAARGRG 453 Rhodobacter sphaeroides
NP_377042   398 VIDPNRKVRISAdvlh-----------------sniNYTIYEGMEVEGWNIMTIRRGEIVYEe--GQVIGKKGSG 453 Sulfolobus tokodaii
EAA67040    428 IWYPTAEQAEAMqagsssrvtmksfqlknemlhhdiDYTPFEGMEFTNWPRYTILRGKLVWDrdgGGVIGGKGDG 502 Aspergillus nidulans ...
ZP_00028774 469 IWNEGGEYPVENtrlh-----------------hnvDYTPYEGMRLTAWPAITLSRGDIVWDg--DRPCGETGRG 524 Burkholderia fungorum
NP_769935   420 LWDPKLKKPIQQadlh-----------------hgaDYTPWEGFDVTGWPVTTVARGRVVYEh--GRIVGDKGAG 475 Bradyrhizobium japoni...
```

Figure S3. Alignment of cyclic amidohydrolase sequences. Metal-interacting amino acids are shown in yellow.

```
ATCC 31783       MTKIIKNGTIVTATDTYEADLLIKDGKIAMIGQHLEEKDAEVIDAKGCYVFPGGIDPHTHLDMPFGGTVTKDDFESGTIA  80
SD1              MTKIIKNGTIVTATDTYEAHLLIKDGKIAMIGQNLEEKGAEVIDAKGCYVFPGGIDPHTHLDMPLGGTVTKDDFESGTIA  80
ATCC 31195       MKKIIKNGTIVTATDTYEADLLIKDGKIAMIGQHLEEKGAEVIDAKGYYVFPGGIDPHTHLDMPFGGTVTKDDFESGTIA  80
NS 1122A         MTKIIKNGTIVTATDTYEADLLIKDGKIAMIGQHLEEKGAEVIDANGCYVFPGGIDPHTHLDMPFGGTVTKDDFESGTIA  80

ATCC 31783       AAFGGTTTIIDFCLTNKGEPLKKAIETWHNKARGKAVIDYGFHLMISEITDEVLEELPKVIEEEGITSFKVFMAYKNVFQ  160
SD1              AAFGGTTTIIDFCLTNKGEPLKKAIETWHNKANGKAVIDYGFHLMISEITDDVLEELPKVLEEEGITSLXVFMAYKNVFQ  160
ATCC 31195       AAFGGTTTIIDFCLTNKGEPLKKAIETWHNKAKGKAVIDYSFHLMISEITDEVLEELPKVIEEEGITSFKVFMAYKNVFQ  160
NS 1122A         AAFGGTTTIIDFCLTNKGEPLKKAIETWHNKAKGKAVIDYGFHLMISEITDDVLEELPKVIAEEGITSFKVFMAYKNVFQ  160

ATCC 31783       ADDGTLYRTLVAAKELGALVMVHAENGDVIDYLTKKALAEGNTDPIYHALTRPPELEGEATGRACQLTELAGSQLYVVHV  240
SD1              ADDGTLYCTLLAAKELGALVMVHAENGDVIDYLTKKALADGNTDPIYHALTRPPELEGEATGRACQLTELAGSQLYVVHV  240
ATCC 31195       ADDGTLYRTLVAAKELGALVMVHAENGDVIDYLTKKALADGNTDPIYHALTRPPELEGEATGRACQLTELAGSQLYVVHV  240
NS 1122A         ADDGTLYRTLVAAKELGALVMVHAENGDVIDYLTKKALAEGNTEPIYHALTRPPEVEGEATGRACQLTELAGSQLYVVHV  240

ATCC 31783       TCAQAVEKIAKARNKGLDVWGETCPQYLVLDQSYLEKPNFEGAKYVWSPPLREKWHQEVLWNALKNGQLQTLGSDQCSFD  320
SD1              TCAQAVEKIAEARNKGLDVWGETCPQYLVLDQSYLEKPNFEGAKYVWSPPLREKWHQEVLWNALKNGQLQTLGSDQCSFD  320
ATCC ATCC 31195  TCAQAVEKIAEARNKGLDVWGETCPQYLVLDQSYLEKPDFEGAKYVWSPPLREKWHQEVLWNALKNGQLQTLGSDQCSFD  320
NS 1122A         TCAQAVEKIAQARNKGLDVWGETCPQYLVLDQSYLEKPDFEGAKYVWSPPLREKWHQEVLWNALKNGQLQTLGSDQCSFD  320

ATCC 31783       FKGQKELGRGDFTKIPNGGPIIEDRVSILFSEGVKKGRITLNQFVDIVSTRIAKLFGLFPKKGTIAVGSDADLVIFDPNI  400
SD1              FKGQKELGRGDFTKIPNGGPIIEDRVSILFSEGVKKGRITLNQFVDIVSTRIAKLFGLFPKKGTIVVGSDADLVIFDPNI  400
ATCC 31195       FKGQKELGRGDFTKIPNGGPMIEDRVRILFSEGVKKGRITLNQFVDIMSTRIAKLFGLFPKKGTIAVGSDADLVIFDPNI  400
NS 1122A         FKGQKELGRGDFTKIPNGGPMIEDRVSILFSEGVKKGRITLNQFVDIMSTRIAKLFGLFPIKGTIAVGSDADLVIFDPDI  400

ATCC 31783       ERVISAETHHMAVDYNAFEGMKVTGEPVSVLCRGEFVVRDKQFVGKPGYGQYLKRAKYGTSTISKQSEELTI        472
SD1              ERVISAETHHMAVDYNAFEGMKVTGEPVSVLCRGEFVVRDKQFVGKPGYGQYLKRAKYGT------------        460
ATCC 31195       ERVISAETHHMAVDYNAFEGMKITGEPVSVLSRGEFVVRDKQFVGKPGYGQYLKRAKYGTLTLSKQDEKLTI        472
NS 1122A         ERVISAETHHMAVDYNAFEGMKVTGEPVSVLSRGEFVVRDKQFVGKPGYGQYLKRAKYGTSKISKQNEKLTI        472
```

Figure S4. D-hydantoinases of G. stearothermophilus strains have high levels of sequence similarity. Metal-interacting amino acids are shown in bold and yellow. .
